# Imperforate Anus

**Published:** 1848-01

**Authors:** 


*Imperforate Anus.*—Dr. Keiller communicated two very interesting cases of imperforate anus from Dr. Lyell, Dundee. In one of the cases Dr. L. had recourse to the measure usually adopted in order to establish a perineal outlet for the fœces. The operation of cutting down upon the gut was not followed by any permanent benefit, although performed thrice during a period of six weeks, about the end of which time, however, some fœculent matter was observed to pass with the urine per urethram, and in a few days afterwards the fœces totally ceased to be emitted by the artificial anus, which soon became obliterated. *The child*

*continued quite well, passing its fœces by the urethra until it was exactly twelve months old,* when convulsions and death supervened in consequence of a complete stoppage, both of urine and fœces having taken place from obstruction of the urethra. On dissection Dr. Lyell discovered that in operating, he had always cut into the urinary bladder, into which a small and imperfect gut was found to terminate, there being no regular rectum, sigmoid flexure, or descending colon present. The obstruction was found to have taken place in the prostatic portion of the urethra, which was completely filled with a substance resembling skins of raisins, coated with brown calcareous deposit. It was somewhat remarkable that during the *first six weeks* of this child's life its urine was passed in a *limpid* stream, without the slightest admixture of fœculent matter, which latter was, throughout that period, evacuated by the opening made by Dr. L. in the perineum, from which moreover no urine was ever observed to pass.

The second case was remarkable from the child having *lived upwards of twelve weeks without any fœcal outlet but that of the mouth.* Dr. Lyell was prevented from attempting the formation of an artificial anus in this case; a post-mortem examination proved, however, that no operation could have been successful, as the blind pouch of the gut was far beyond the reach of the knife. The anatomical peculiarity in this case consisted in the colon terminating in a large globular cul de sac, which was found floating loosely among the other intestines in the umbilical region. Dr. Keiller exhibited preparations from the above cases. Both were male children.

